# EP300 single nucleotide polymorphism rs20551 correlates with prolonged overall survival in diffuse large B cell lymphoma patients treated with R-CHOP

**DOI:** 10.1186/s12935-017-0439-1

**Published:** 2017-07-14

**Authors:** Jiao Li, Ning Ding, Xiaogan Wang, Lan Mi, Lingyan Ping, Xuan Jin, Yalu Liu, Zhitao Ying, Yan Xie, Weiping Liu, Yuqin Song, Jun Zhu

**Affiliations:** 1 Key Laboratory of Carcinogenesis and Translational Research (Ministry of Education), Department of Lymphoma, Peking University Cancer Hospital & Institute, No. 52 Fucheng Road, Haidian District, Beijing, 100142 People’s Republic of China; 2 Key Laboratory of Carcinogenesis and Translational Research (Ministry of Education), Peking University Cancer Hospital & Institute, No. 52 Fucheng Road, Haidian District, Beijing, 100142 People’s Republic of China; 3 Department of Internal Medicine Oncology, Peking University First Hospital, No. 1 Xishiku Road, Xicheng District, Beijing, 100034 People’s Republic of China

**Keywords:** Diffuse large B-cell lymphoma, EP300, rs20551, Single nucleotide polymorphism, R-CHOP chemotherapy

## Abstract

**Background:**

Rituximab combined with cyclophosphamide, doxorubicin, vincristine and prednisone (CHOP) is used as standard frontline regimen for diffuse large B-cell lymphoma (DLBCL). The landscape of somatic mutations in DLBCL revealed that inactivation of EP300 plays an important role in lymphomagenesis. A common EP300 single nucleotide polymorphism (SNP) rs20551 results in the substitution of valine for isoleucine at codon 997 close to the Bromodomain. However, the association between SNP rs20551 and clinical prognosis in DLBCL patients treated with R-CHOP is unknown.

**Methods:**

In this study we analyzed the EP300 SNP rs20551 and prognosis of 226 DLBCL patients who treated with R-CHOP or R-CHOP-like regimes from 2002 to 2013. Determination of the EP300 SNP rs20551 from genomic DNA was obtained by Sanger chain termination sequencing.

**Result:**

In this study, the frequency of the A and G allele of the EP300 SNP rs20551 in 226 patients were 92.5 and 7.5%, respectively. We did not observe obvious correlation between patients’ disease features and the EP300 SNP rs20551. But the patients with genotype AA had a higher 5-year overall survival rate than those with genotype GA (77.0% vs. 64.7%, p = 0.045). Furthermore, multivariate Cox regression analysis showed that the GA genotype of EP300 SNP rs20551 was an independent poor prognostic factor for DLBCL patients treated with Rituximab-chemotherapy (p = 0.009, HR 2.956, 95% CI 1.315–6.645).

**Conclusion:**

This study suggests that EP300 SNP rs20551 might be a useful biomarker to predict the long-term outcome of R-CHOP in DLBCL patients.

## Background

Diffuse large B-cell lymphoma (DLBCL) is the most common subtype of B-cell non-Hodgkin’s lymphoma (NHL) and can be classified into two cell-of-origin molecular subtypes: germinal center B-cell (GCB) and activated B-cell (ABC) types [1]. DLBCL is an aggressive and heterogeneous disease generally treated with the anthracycline-based chemotherapy regimen of CHOP (cyclophosphamide, doxorubicin, vincristine and prednisone) [2, 3]. The CD20 antibody rituximab is commonly used to treat many types of CD20-positive NHL, including DLBCL. The combination R-CHOP regimen dramatically improves outcome and prognosis, which is considered as the standard treatment for DLBCL [4, 5]. However, approximately 30% of patients do not respond or develop resistance to this immunotherapy, which remains a major cause of morbidity and mortality [6]. The prognostic outcome of DLBCL depends on patient age, clinicopathological features, and genetic factors. The International Prognostic Index (IPI) is identified as the most common and useful factor to predict the prognosis of NHL, including DLBCL [7]. However, the efficiency of IPI could be enhanced by inclusion of additional prognostic markers. Therefore, development of novel prognostic factors based on the biology of DLBCL is an unmet clinical need.

EP300, also known as E1A binding protein p300, is encoded by the EP300 gene, which is located on chromosome 22q13.2. EP300 is a member of the histone acetyltransferase (HAT) family [8, 9] that regulates transcription via catalyzing post-translational acetylation of lysine residues. EP300 plays an essential role in regulating vital cellular processes such as the cell cycle and apoptosis by modulating chromatin structure through its own HAT activity and by interacting with transcription factors and other histone acetylators [10, 11]. EP300 knockout results in early embryonic lethality and germline mutation of EP300 causes Rubinstein–Taybi syndrome, which is characterized by increased predisposition to childhood malignancy [12]. The role for EP300 in cancer is implied by the fact that it is targeted by oncoproteins, it is fused to MLL in leukemia, and two missense sequence alterations in EP300 have been identified in epithelial malignancy [13–16]. In addition, somatic mutations in EP300 have been found in gastric cancer, colon cancer, acute myeloid leukemia and glioblastoma [16–18]. Next-generation whole-exome sequencing analysis has indicated that inactivating mutations of EP300 play a haploinsufficient role in the pathogenesis of malignancies derived from B lymphocytes [19]. EP300 mutation represents a common event that has been identified in 10% of DLBCL patients. In the EP300 gene, several mutations have been observed in the catalytic HAT domains. The mutant proteins in the HAT domain have lost their normal acetyltransferase activity, which causes significant pathological and developmental phenotypes and affects the therapeutic response of tumor cells [19]. rs20551 (A/G) is a single nucleotide polymorphism (SNP) of EP300 gene, which results in an amino acid alternation from isoleucine to valine at position 997. Analysis of Ensembl data [20] has shown that this amino acid is located upstream of the bromodomain and may play an important role in HAT activity, suggesting that rs20551 may influence the protein function of EP300.

The impact of the rs20551 polymorphism of EP300 gene on DLBCL prognosis has not been fully elucidated. Here, this is the first study to investigate the association between EP300 SNP rs20551 and DLBCL prognosis. Survival analysis revealed that EP300 SNP rs20551 was significantly correlated with clinical outcome, indicating that rs20551 could be an independent predictor for prognosis in DLBCL patients treated with R-chemotherapy.

## Methods

### Patient characteristics

From December 2002 to November 2013, 226 patients with pathologically diagnosed DLBCL who received R-CHOP or R-CHOP-like chemotherapy (e.g. R-COP, R-CCOP, R-CHO or R-CHOPE) as a frontline regimen were recruited for this retrospective study from Peking University Cancer Hospital. In all of 226 patients, fourteen patients have received autologous stem-cell transplantation. Peripheral blood specimens from all DLBCL patients were obtained before initial therapy. R-CHOP chemotherapy was administrated as follows: one course of chemotherapy consisted of an intravenous infusion of 750 mg/m^2^ cyclophosphamide, 50 mg/m^2^ doxorubicin, and 2 mg vincristine, and oral administration of 100 mg prednisone on days 1–5, which was repeated every 3 weeks. Rituximab 375 mg/m^2^ was infused over 4–6 h on day 1 before CHOP or CHOP-like chemotherapy was started. The response to R-CHOP was evaluated after completion of 2–3 courses of treatment and 1–2 months after completion of all therapy, then every 3 months for the first year and every 6 months thereafter until progression.

We obtained all the clinical and pathological data by medical record review, including gender, age, tumor stage, histological subtype, B symptoms, IPI score, Eastern Cooperative Oncology Group performance status (ECOG PS), lymph node or extranodal invasion, hematological examination, therapeutic regimens, and curative effect. Informed consent was obtained from all individual participants included in the study. This retrospective study was approved by our Institutional Review Board.

### DNA extraction and genotyping

Genomic DNA was isolated from whole blood with the QIAmp DNA Mini Kit (Qiagen, Hilden, Germany) and stored at −20 °C. Genomic DNA was diluted in nuclease-free water to a final stock concentration of 30 ng/μl, and 1 μl DNA stock was used in each PCR. The primer for rs20551 was forward 5′-TTGCTGAGAAGCAGCCTTCC-3′ and reverse 5′-CTTGGCTGGTCTTCCTCCTC-3′. PCR conditions were programmed on a thermo cycler (Gene Cycler, Bio-Rad, Hercules, CA, USA) as follows: denaturation at 94 °C for 5 min, followed by 35 cycles of 94 °C for 30 s, 60 °C for 40 s, 72 °C for 40 s, followed by 72 °C for 7 min. Amplified products were analyzed by gel electrophoresis on 2% agarose gels, and were sequencing using ABI 3730XL Avant Genetic Analyzer (Applied Biosystems, Carlsbad, CA, USA). Finally, the sequences were analyzed with the Seqman software (DNASTAR, Madison, WI, USA).

### Statistical analysis

Overall survival (OS) was measured from the date of diagnosis to the date of death or last follow-up for those who were alive at the time of analysis. Progression-free survival (PFS) was calculated from the date of diagnosis to the date of disease progression or to the date of the last follow-up for those who were alive at the time of analysis. The clinical characteristics and response rate of the patients were compared using Chi square tests. The Kaplan–Meier method was used to construct survival curves, and results were compared using a log-rank test. The Cox regression model was used to evaluate the prognostic factors. p < 0.05 was considered to indicate statistically significant differences. IBM SPSS Statistics version 20.0 was used for all statistical analysis. The survival curves were displayed using GraphPad Prism 6 software.

## Results

### Patient characteristics

The general characteristics of the 226 DLBCL patients (108 male and 118 female) in this study are summarized in Table 1. The median age at diagnosis was 57 years (range, 15–90 years). One hundred and forty-seven patients (65.0%) exhibited B symptom, 129 (57.1%) patients were in stages 3 or 4, and 75 (33.2%) patients were at intermediate-to-high or high risk according to the international prognostic index (IPI) scores. One hundred and fifty-three (73.9%) patients were classified into non-GCB subtype. Bone marrow was involved by lymphoma in 11 patients (4.9%) at diagnosis. Two hundred and twenty-six patients received R-CHOP or R-CHOP like treatment as a frontline regimen, and the clinical efficacy was evaluated in this retrospective study.

**Table 1:** DLBCL patients’ characteristics and their correlations with the genotype of EP300 SNP rs20551

Clinical parameters	No.	Genotype		p	Clinical parameters	No.	Genotype		p
		AA	GA				AA	GA	
Gender					Stage				
Male	108	91	17	0.779	I–II	97	85	12	0.330
Female	118	101	17		III–IV	129	107	22	
Age					IPI score				
≤60	144	123	21	0.797	0–2	151	128	23	0.911
>60	82	69	13		3–5	75	64	11	
B symptoms					Ki67				
Positive	147	126	21	0.663	≤75%	101	90	11	0.158
Negative	79	66	13		>75%	113	93	20	
LDH					Subtype				
Positive	107	94	13	0.248	GCB	54	46	8	0.969
Negative	119	98	21		non-GCB	153	130	23	
β2-MG					Extra nodal site				
Positive	56	46	10	0.385	≤1	159	133	26	0.420
Negative	160	139	21		>1	66	58	8	
ESR					HBV infection				
Positive	133	115	18	0.936	Positive	110	93	17	0.764
Negative	79	68	11		Negative	114	98	16	
ECOG									
0–1	208	178	30	0.313					
2–3	17	13	4						

*LDH* lactate dehydrogenase, *MG* microglobulin, *ESR* erythrocyte sedimentation rate, *ECOG* Eastern Cooperative Oncology Group, *IPI* International Prognostic Index, *GCB* germinal center B cell subtype, *HBV* hepatitis-B virus

### EP300 SNP rs20551

The genotype of EP300 SNP rs20551 in 226 patients was shown in Table 2. One hundred and ninety-two (85.0%) patients carried the homozygous AA genotype, 34 (15.0%) patients had the heterozygous GA genotype, and no GG genotype was detected. The frequency of the A allele in 226 patients was 92.5%, and the frequency of the G allele was 7.5%. The genotype distribution of SNP rs20551 in the DLBCL population included in our study was in Hardy–Weinberg equilibrium (Fisher’s exact test, p = 1.000).

**Table 2:** Genotype and allele frequency of EP300 SNP rs20551 in 226 DLBCL patients

	Frequency	Count
Genotype		
AA	0.850	192
GA	0.150	34
GG	0	0
Allele		
A	0.925	418
G	0.075	34

### Impact of EP300 SNP rs20551 on the efficacy of R-chemotherapy

Of all the 226 patients evaluable for response to R-CHOP or R-CHOP like treatment, the overall response rate (ORR) was 85.8% (194 of 226 patients), including a complete response (CR) rate of 67.3% (152 of 226 patients) and a partial response (PR) rate of 18.6% (42 of 226 patients). However, as shown in Table 3, no correlation between SNP rs20551 and the efficacy of R-chemotherapy was observed.

**Table 3:** Clinical response to R-CHOP therapy according to the genotype of EP300 SNP rs20551

Response	Genotype		p
	AA	GA	
All patients			
CR	130	22	0.731
PR + PD + SD	62	12	
OR	163	31	0.333
PD + SD	29	3	
Non-GCB subtype			
CR	84	12	0.255
PR + PD + SD	46	11	
OR	110	21	0.399
PD + SD	20	2	
GCB subtype			
CR	36	8	0.144
PR + PD + SD	10	0	
OR	39	8	0.237
PD + SD	7	0	

*CR* complete response, *PR* partial response, *OR* overall response, *SD* stable disease, *PD* progressive disease

### Survival analyses according to the genotype of EP300 SNP rs20551

**Fig. 1:**
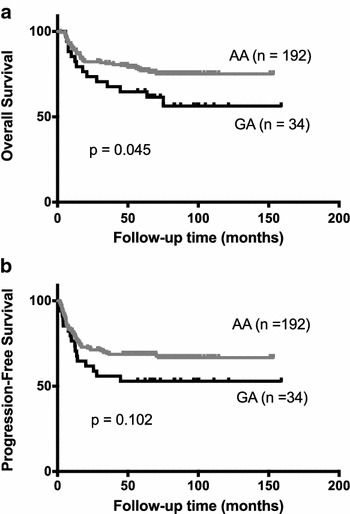
Kaplan–Meier curves for overall survival and progression-free survival in DLBCL patients according to the genotype of EP300 SNP rs20551

All 226 patients were evaluated for overall survival (OS) and progression-free survival (PFS). After a median follow-up time of 63.5 months (range 2.0–158.9 months), 78 (34.5%) patients relapsed or progressed and 59 (26.1%) patients died. As shown in Fig. 1a, the patients with genotype AA had a higher 5-year OS rate than those with genotype GA (77.0% vs. 64.7%, p = 0.045), which might indicate the role of EP300 SNP rs20551 as a biomarker to predict the long-term outcome of R-CHOP or R-CHOP like treatment. A 5-year PFS rate of 68.6% was observed in the AA patients, compared with 52.9% in the GA patients; but this higher PFS rate did not reach statistical significance (p = 0.102, Fig. 1b).

### Univariate and multivariate analyses

In the univariate analysis, the following variables on OS were evaluated, including gender (male vs. female), age (≤60 vs. >60 years), stage (stages 1, 2 vs. 3, 4), IPI score (0–2 vs. 3–5), B symptoms (positive vs. negative), subtype (GCB vs. non-GCB), Ki67 (≤75 vs. >75%), LDH level (normal vs. abnormal), β2-microglobulin level (normal vs. abnormal), ESR (normal vs. abnormal), ECOG (0–1 vs. 2–3), extra nodal involvement (≤1 site vs. >1 sites), HBV infection (positive vs. negative), clinical response (OR vs. PD + SD), and EP300 SNP rs20551 (AA vs. GA). The analysis has shown that age > 60 years (p = 0.018), higher stage (p < 0.001), abnormal B symptom (p = 0.016), abnormal LDH level (p < 0.001), abnormal β2-microglobulin level (p = 0.001), higher IPI score (p < 0.001), PD + SD (p < 0.001), extra nodal involvement >1 sites (p = 0.001), and the GA genotype of EP300 rs20551 (p = 0.048) were significantly associated with poor OS. Moreover, as shown in Table 4, multivariate Cox regression analysis displayed that the GA genotype of EP300 SNP rs20551 also was an independent poor prognostic factor for OS (p = 0.009, HR 2.956, 95% CI 1.315–6.645).

**Table 4:** Multivariate analysis of EP300 SNP rs20551 on survival in 226 DLBCL patients

Variable	Hazard ratio	95% CI	p
GA/AA	2.956	1.315–6.645	0.009
B symptom	0.502	0.271–0.928	0.028
β2-MG	2.239	1.201–4.175	0.011
IPI	2.435	1.362–4.354	0.003
Clinical response	0.06	0.030–0.120	<0.001

## Discussion

The acetylation of histone lysine residues is a dynamic process, which is regulated by the balance between the activity of acetyltransferases and deacetylases. Acetylation of lysine residues on histone weakens its interaction with DNA, thereby conferring a more open chromatin structure and allowing active transcription. EP300 acts as an intrinsic HAT, which is also a transcriptional coactivator, and plays a critical role in tumorigenesis [21, 22]. Thus, gene mutations influencing the structure of EP300 are found in several types of cancer, including BCL [19, 23]. However, despite the clearly important role for EP300 mutation in lymphomagenesis, the exact mechanisms by which they promote malignant transformation remains largely undefined. Previous studies have shown that EP300 mutants have reduced HAT activity to acetylate BCL6 proto-oncogene and P53 anti-oncogene [19], disrupting the balance between the activities of these two genes, which is critical for the regulation of DNA damage response in mature GCB cells [24, 25].

The rs20551 polymorphism of EP300 gene is located in the coding sequence in exon 15, in the chromosomal region 22q13.2, and codes for a missense change (I > V), which could influence the protein structure and function of EP300. A study in Hispanic Americans has demonstrated that rs20551 gene variant in EP300 plays an important role in childhood acute lymphoblastic leukemia [26]. The influence of gene variants in rs20551 on clinical outcome in recipients who diagnosed with a hematological malignancy that received HLA-identical sibling allo-stem cell transplantation has been investigated. Patients with AA gene variant in rs20551 had a lower relapse rate and better disease-free survival and OS. The study showed that rs20551 in EP300 was associated with different expression in genes involved in innate immunity and the cell cycle. Patients with the AA gene variant had lower interleukin-2 production, higher expression of genes involved in innate immunity and the cell cycle, and lower expression of p53 [27]. However, none of the rs20551 genetic variants has been previously reported in association studies with lymphoma prognosis, including DLBCL. In this study, we analyzed the relationship between rs20551 genotype and clinical features and prognosis in DLBCL patients and found that patients with the homozygous A allele in rs20551 had longer OS than the G allele carriers had. However, it is unclear exactly how EP300 polymorphism in rs20551 affects survival of patients with DLBCL after R-CHOP chemotherapy. Our results showed that there was no association between rs20551 genotype and curative effect. We hypothesize that the genotype change in EP300 polymorphism might affect the HAT activity by causing amino acid substitution, which disrupts the balance between acetylation and deacetylation in the tumor microenvironment and influences disease progression.

SNPs are variants in a single nucleotide that occur in individual genomes and remain consistent throughout a person’s lifetime. For population diversity, allele frequency data about EP300 SNP rs20551 can be obtained in the NCBI SNP database, which demonstrates that the frequency of allele G for EP300 SNP rs20551 is 5.36% in normal East Asian individuals. However, our data showed a minor difference in distribution for allele G frequency between the control group of East Asians and DLBCL patients. Our analysis indicated that the frequency of allele G in rs20551 was 7.5% in DLBCL patients, which was higher than that in healthy East Asians. The change from allele A to G in rs20551 results in amino acid alteration, which might influence the protein function of EP300. This difference in allele frequency might explain the process of lymphomagenesis.

The EP300/CREB-binding protein (CREBBP) complex acts to acetylate multiple lysine residues on histones. EP300/CREBBP is involved in a wide range of cellular activities including proliferation, cell cycle regulation, apoptosis, differentiation and DNA repair [17, 28]. Mutation in this complex in lymphoma has been described, showing that about 39% of DLBCL and 41% of follicular lymphoma patients have genomic deletions and/or somatic mutations that inactivate or remove the acetyltransferase domain of EP300 and CREBBP [19]. EP300/CREBBP also targets many other transcriptional factors that are significant in the development of BCL, such as p53, BCL6 and c-MYC [19, 29–31]. Moreover, the CREBBP and EP300 coactivator associates with various transcription factors that play a critical role in the immune response [21]. Since all these significant biological functions are regulated by EP300 gene, it is believed that a constitutional variant in this gene could significantly influence the clinical outcome of DLBCLs.

The genetic variants of HATs generally indicate extensive deregulation of histone acetylation in B-cell NHL. From a therapeutic perspective, epigenetic therapies such as histone deacetylase (HDAC) inhibitors could provide the opportunity to re-establish physiological acetylation levels, which would be a potential therapeutic intervention for DLBCL patients with genetic variants of EP300. These compounds such as vorinostat and panobinostat have shown some efficacy in hematological malignancy, including DLBCL, follicular lymphoma and mantle cell lymphoma [32–34]. However, previous studies could not confirm the association between the genetic variants of EP300 and curative response to HDAC inhibitors, possibly due to their retrospective approach and small number of samples. Therefore, further investigations are needed to find factors that predict the clinical efficacy of HDAC inhibitors, such as a prospective analysis of constitutional gene variants of EP300 and/or CREBBP in randomized clinical trials.

In summary, our results revealed that the GA genotype of EP300 SNP rs20551 as an independent poor prognostic factor in DLBCL patients. To the best of our knowledge, this is the first study to explore the association between EP300 SNP rs20551 and prognosis of DLBCL. Our study might help to improve prediction of long-term outcome in patients with DLBCL treated with R-CHOP, or prompt the use of HDAC inhibitors for further treatment, especially for patients with the heterozygous GA genotype at EP300 SNP rs20551.

## Conclusion

In this retrospective study, we find that the DLBCL patients with genotype AA of EP300 SNP rs20551 show better survival than those with genotype GA after R-CHOP or R-CHOP like treatment. Therefore, EP300 SNP rs20551 could be considered as an independent biomarker to predict the long-term outcome of R-chemotherapy in DLBCL patients.

## Abbreviations

DLBCL: diffuse large B-cell lymphoma
NHL: non-Hodgkin’s lymphoma
GCB: germinal center B-cell
ABC: activated B-cell
CHOP: cyclophosphamide, doxorubicin, vincristine and prednisone
R: rituximab
IPI: International Prognostic Index
HAT: histone acetyltransferase
HDAC: histone deacetylase
SNP: single nucleotide polymorphism
ECOG: Eastern Cooperative Oncology Group
OS: overall survival
PFS: progression-free survival
OR: overall response
CR: complete response
PR: partial response
SD: stable disease
PD: progressive disease
LDH: lactate dehydrogenase
MG: macroglobulin
ESR: erythrocyte sedimentation rate
HBV: hepatitis-B virus
AA: AA genotype of rs20551
GA: GA genotype of rs20551

## References

[1] Alizadeh AA, Eisen MB, Davis RE, Ma C, Lossos IS, Rosenwald A, Boldrick JC, Sabet H, Tran T, Yu X (2000). Distinct types of diffuse large B-cell lymphoma identified by gene expression profiling. Nature.

[2] Coiffier B, Thieblemont C, Van Den Neste E, Lepeu G, Plantier I, Castaigne S, Lefort S, Marit G, Macro M, Sebban C (2010). Long-term outcome of patients in the LNH-98.5 trial, the first randomized study comparing rituximab-CHOP to standard CHOP chemotherapy in DLBCL patients: a study by the Grouped’Etudes des Lymphomes de l’Adulte. Blood.

[3] Pfreundschuh M, Schubert J, Ziepert M, Schmits R, Mohren M, Lengfelder E, Reiser M, Nickenig C, Clemens M, Peter N (2008). Six versus eight cycles of bi-weekly CHOP-14 with or without rituximab in elderly patients with aggressive CD20 + B-cell lymphomas: a randomised controlled trial (RICOVER-60). Lancet Oncol.

[4] Sehn LH, Donaldson J, Chhanabhai M, Fitzgerald C, Gill K, Klasa R, MacPherson N, O’Reilly S, Spinelli JJ, Sutherland J (2005). Introduction of combined CHOP plus rituximab therapy dramatically improved outcome of diffuse large B-cell lymphoma in British Columbia. J Clin Oncol.

[5] Habermann TM, Weller EA, Morrison VA, Gascoyne RD, Cassileth PA, Cohn JB, Dakhil SR, Woda B, Fisher RI, Peterson BA (2006). Rituximab-CHOP versus CHOP alone or with maintenance rituximab in older patients with diffuse large B-cell lymphoma. J Clin Oncol.

[6] Friedberg JW (2011). Relapsed/refractory diffuse large B-cell lymphoma. Hematol Am Soc Hematol Educ Progr.

[7] Rossi G, Donisi A, Casari S, Re A, Cadeo G, Carosi G (1999). The International Prognostic Index can be used as a guide to treatment decisions regarding patients with human immunodeficiency virus-related systemic non-Hodgkin lymphoma. Cancer.

[8] Ogryzko VV, Schiltz RL, Russanova V, Howard BH, Nakatani Y (1996). The transcriptional coactivators p300 and CBP are histone acetyltransferases. Cell.

[9] Bannister AJ, Kouzarides T (1996). The CBP co-activator is a histone acetyltransferase. Nature.

[10] Brook CL, Gu W (2011). The impact of acetylation and deacetylation on the p53 pathway. Protein Cell..

[11] Shigeno K, Yoshida H, Pan L, Luo JM, Fujisawa S, Naito K, Nakamura S, Shinjo K, Takeshita A, Ohno R (2004). Disease-related potential of mutations in transcriptional cofactors CREB-binding protein and p300 in leukemias. Cancer Lett.

[12] Fergelot P, Van Belzen M, Van Gils J, Afenjar A, Armour CM, Arveiler B, Beets L, Burglen L, Busa T, Collet M (2016). Phenotype and genotype in 52 patients with Rubinstein-Taybi syndrome caused by EP300 mutations. Am J Med Genet Part A.

[13] Arany Z, Newsome D, Oldread E, Livingston DM, Eckner R (1995). A family of transcriptional adaptor proteins targeted by the E1A oncoprotein. Nature.

[14] Lundblad JR, Kwok RP, Laurance ME, Harter ML, Goodman RH (1995). Adenoviral E1A-associated protein p300 as a functional homologue of the transcriptional co-activator CBP. Nature.

[15] Ida K, Kitabayashi I, Taki T, Taniwaki M, Noro K, Yamamoto M, Ohki M, Hayashi Y (1997). Adenoviral E1A-associated protein p300 is involved in acute myeloid leukemia with t(11;22)(q23;q13). Blood.

[16] Muraoka M, Konishi M, Kikuchi-Yanoshita R, Tanaka K, Shitara N, Chong JM, Iwama T, Miyaki M (1996). p300 gene alterations in colorectal and gastric carcinomas. Oncogene.

[17] Giles RH, Peters DJ, Breuning MH (1998). Conjunction dysfunction: CBP/p300 in human disease. Trends Genet.

[18] Borrow J, Stanton VP, Andresen JM, Becher R, Behm FG, Chaganti RS, Civin CI, Disteche C, Dubé I, Frischauf AM (1996). The translocation t(8;16)(p11;p13) of acute myeloid leukaemia fuses a putative acetyltransferase to the CREB-binding protein. Nat Genet.

[19] Pasqualucci L, Dominguez-Sola D, Chiarenza A, Fabbri G, Grunn A, Trifonov V, Kasper LH, Lerach S, Tang H, Ma J (2011). Inactivating mutations of acetyltransferase genes in B-cell lymphoma. Nature.

[20] Ensembl Database. European Bioinformatics Institute and Wellcome Trust Sanger Institute. Cambridge. 2000. http://asia.ensembl.org. Accessed July 2000.

[21] Weaver BK, Kumar KP, Reich NC (1998). Interferon regulatory factor 3 and CREB-binding protein/p300 are subunits of double-stranded RNA-activated transcription factor DRAF1. Mol Cell Biol.

[22] Liu X, Wang L, Zhao K, Thompson PR, Hwang Y, Marmorstein R, Cole PA (2008). The structural basis of protein acetylation by the p300/CBP transcriptional coactivator. Nature.

[23] Gayther SA, Batley SJ, Linger L, Bannister A, Thorpe K, Chin SF, Daigo Y, Russell P, Wilson A, Sowter HM (2000). Mutations truncating the EP300 acetylase in human cancers. Nat Genet.

[24] Phan RT, Dalla-Favera R (2004). The BCL6 proto-oncogene suppresses p53 expression in germinal-centre B cells. Nature.

[25] Phan RT, Saito M, Basso K, Niu H, Dalla-Favera R (2005). BCL6 interacts with the transcription factor Miz-1 to suppress the cyclin-dependent kinase inhibitor p21 and cell cycle arrest in germinal center B cells. Nat Immunol.

[26] Piwkham D, Gelfond JA, Rerkamnuaychoke B, Pakakasama S, Rebel VI, Pollock BH, Winick NJ, Collier AB, Tomlinson GE, Beuten J (2011). Multilocus association of genetic variants in MLL, CREBBP, EP300, and TOP2A with childhood acute lymphoblastic leukemia in Hispanics from Texas. Cancer Epidemiol Biomark Prev.

[27] Martín-Antonio B, Álvarez-Laderas I, Cardesa R, Márquez-Malaver F, Baez A, Carmona M, Falantes J, Suarez-Lledo M, Fernández-Avilés F, Martínez C (2012). A constitutional variant in the transcription factor EP300 strongly influences the clinical outcome of patients submitted to allo-SCT. Bone Marrow Transpl.

[28] Goodman RH, Smolik S (2000). CBP/p300 in cell growth, transformation, and development. Genes Dev.

[29] Gu W, Roeder RG (1997). Activation of p53 sequence-specific DNA binding by acetylation of the p53 C-terminal domain. Cell.

[30] Tang Y, Zhao W, Chen Y, Zhao Y, Gu W (2008). Acetylation is indispensable for p53 activation. Cell.

[31] Vervoorts J, Lüscher-Firzlaff JM, Rottmann S, Lilischkis R, Walsemann G, Dohmann K, Austen M, Lüscher B (2003). Stimulation of c-MYC transcriptional activity and acetylation by recruitment of the cofactor CBP. EMBO Rep.

[32] Kirschbaum M, Frankel P, Popplewell L, Zain J, Delioukina M, Pullarkat V, Matsuoka D, Pulone B, Rotter AJ, Espinoza-Delgado I (2011). Phase II study of vorinostat for treatment of relapsed or refractory indolent non-Hodgkin’s lymphoma and mantle cell lymphoma. J Clin Oncol.

[33] Assouline SE, Nielsen TH, Yu S, Alcaide M, Chong L, MacDonald D, Tosikyan A, Kukreti V, Kezouh A, Petrogiannis-Haliotis T (2016). Phase 2 study of panobinostat with or without rituximab in relapsed diffuse large B-cell lymphoma. Blood.

[34] Ogura M, Ando K, Suzuki T, Ishizawa K, Oh SY, Itoh K, Yamamoto K, Au WY, Tien HF, Matsuno Y (2014). A multicentre phase II study of vorinostat in patients with relapsed or refractory indolent B-cell non-Hodgkin lymphoma and mantle cell lymphoma. Br J Haematol.

